# Supplementary material for: Spatial and multilevel analysis of sanitation service access and related factors among households in Ethiopia: Using 2019 Ethiopian national dataset
Source: PLOS Glob Public Health. 2023 Apr 4;3(4):e0001752. doi: 10.1371/journal.pgph.0001752 (PMC10072458; doi:10.1371/journal.pgph.0001752)
Supplement: Attachment — Submitted filename: Point by point response.docx [file pgph.0001752.s001.docx]

**Dear editorial board member (s) of** **Plos global public health journal**

We the authors have been recalled to revise the manuscript entitled ***Spatial and Multilevel Analysis of Sanitation Service Access and Related Factors in Ethiopia: Using 2019 Ethiopian national data*** *which is* identified by submission ID PGPH-D-22-01942 under Plos global public health journal, which was submitted for publication.

Thank are very thankful for receiving comments from editors and reviewers. Authors are very happy in receiving constructive and valuable comments for the advancement of the manuscript.

Please be informed that we had been facing a problem of matching the line numbers mentioned by reviewer 2' with the line number of the submitted manuscript. All the rest was fine, if we had been going wrongly, please let us know.

Thank you very much all editor (s), and reviewer.

Yours sincerely,

Correspondence author

**Point-by-point response**

Journal Requirements:

1. Please provide separate figure files in .tif or .eps format only and remove any figures embedded in your manuscript file. Please also ensure that all files are under our size limit of 10MB.

**Authors:** Thank you so much, all the figures are provided in .tiff file format and figures were removed from the main manuscript.

2. We do not publish any copyright or trademark symbols that usually accompany proprietary names, eg  ©, ®, ™  (e.g. next to drug or reagent names). Please remove all instances of trademark/copyright symbols throughout the text, including ™ on page 22.

**Authors:** Thank you so much. We have to pay attention to addressing such issues. If not well, please support us in doing.

3. Your manuscript is missing the following sections: Introduction. Please ensure these are present, and in the correct order, and that any references to subheadings in your main text are correct. An outline of the required sections can be consulted in our submission guidelines.

**Authors:** Thank you so much, we have made corrections on it and related issues.

4. We have noticed that you have uploaded Supporting Information files, but you have not included a list of legends. Please add a full list of legends for your Supporting Information files after the references list.

**Authors:** Thank you so much. We have added the legends for the supporting information.

5. Figs 1-4: please (a) provide a direct link to the base layer of the map (i.e., the country or region border shape) and ensure this is also included in the figure legend; and (b) provide a link to the terms of use / license information for the base layer image or shapefile. We cannot publish proprietary or copyrighted maps (e.g. Google Maps, Mapquest) and the terms of use for your map base layer must be compatible with our CC-BY 4.0 license.

Note: if you created the map in a software program like R or ArcGIS, please locate and indicate the source of the base map shapefile onto which data has been plotted.

**Authors:** Thank you so much. The source of all the data source are presented in the main manuscript as well as source for shape file is described in each map

Reviewers' comments:

**Comments to the Author**
1. Does this manuscript meet PLOS Global Public Health’s [publication criteria](https://journals.plos.org/globalpublichealth/s/criteria-for-publication)? Is the manuscript technically sound, and do the data support the conclusions? The manuscript must describe methodologically and ethically rigorous research with conclusions that are appropriately drawn based on the data presented.

Reviewer #1: Yes

Reviewer #2: Partly

**Authors:** Thank you so much. Improvement on methodology, conclusions and all over the manuscript have been done.

2. Has the statistical analysis been performed appropriately and rigorously?

Reviewer #1: Yes

Reviewer #2: No

**Authors:** Thank you so much all. Improvement have been done on the analysis especially spatial analysis section, and all analysis results.

3. Have the authors made all data underlying the findings in their manuscript fully available (please refer to the Data Availability Statement at the start of the manuscript PDF file)?

The [PLOS Data policy](https://journals.plos.org/globalpublichealth/s/data-availability) requires authors to make all data underlying the findings described in their manuscript fully available without restriction, with rare exception. The data should be provided as part of the manuscript or its supporting information, or deposited to a public repository. For example, in addition to summary statistics, the data points behind means, medians and variance measures should be available. If there are restrictions on publicly sharing data—e.g. participant privacy or use of data from a third party—those must be specified.

Reviewer #1: Yes

Reviewer #2: Yes

**Authors:** Thank you so much all. We have made substantial improvement in the revised manuscript.

4. Is the manuscript presented in an intelligible fashion and written in standard English?

PLOS Global Public Health does not copyedit accepted manuscripts, so the language in submitted articles must be clear, correct, and unambiguous. Any typographical or grammatical errors should be corrected at revision, so please note any specific errors here.

Reviewer #1: No

Reviewer #2: No

**Authors:** Thank you so much all for your concerns. We have made substantial improvement on grammars and spelling in the whole manuscript.

5. Review Comments to the Author

Reviewer #1: “Four out of ten (38.1%) and nearly one-fifth (23.3%) of households were from the Oromia and SNNPR regions, respectively”.
This sentence is not clear it is difficult to catch all the paragraphs. The remaining paragraph talks about Oromia and SNNPR only? Or about Ethiopia?

**Authors:** It is about Ethiopia. Of the total respondents at a household level, 38.1% and 23.3% of respondents were from Oromia and SNNPR regions, respectively. The remaining respondents were from others regions. The detail about this issue is presented in table 1 in the main manuscript.
Reviewer #1: Paragraph line number three says “open defecation being preferable due to unpleasant odors and heat from latrines”. What type of heat can be generated from latrine? Please explain it well.

**Authors:** Thank you! Improvement have been made on it in the revised manuscript.

Reviewer #1: 6th line of the paragraph, the word should be used. It looks like an obligation. If it is a kind of recommendation, please use another better word that makes it a recommendation.

**Authors:** Thank you very much for this issue. In line with your direction improvement have been made to the whole manuscript.
Reviewer #1: The absence of page and line numbers makes it difficult to indicate the exact location of major improvement areas in this manuscript.

**Authors:** Thank you very much, dear!! We have assigned the page number and line number in the revised manuscript.
Reviewer #1: Define the SNNPR abbreviation upon the first appearance in the text.

**Authors:** Thank you, dear!! It is about South Nations Nationality and People’s region. So, we have defined it at the first appearance in the manuscript, in the abstract section of the manuscript.

Reviewer #2: The paper is based on the spatial distribution pattern and associated factors with lack of access to sanitation services in Ethiopia, based on census data.
Due to the demonstrated impact of lack of basic sanitation access on public health, especially in underdeveloped countries, this paper stands out in addressing the problem and identifying areas where these deficits are increased and are major factors that lead to health inequities. Recognizing these areas would improve the cost-benefit access interventions, increasing the population reached by the interventions in countries where resources are extremely limited.
Despite this, the manuscript presents several issues that should be reviewed.
Three different spatial methods were used: Moran I, interpolation, and scan statistics. But was not explained a clear conceptual framework that shows why the use of each of them was and which particular outcome was expected.
Many information is just of local relevance, and with little or not relevance to other areas than Ethiopia. This is a global journal, so explain more in detail about socio-cultural features could help the reader to better visualize the problematic.
The manuscript is based in the EMDHS data, but some results and methodology is different. Please explain why those differences.
There is not a clear conceptual framework in the inclusion of the independent variables. Given the spatial dependency is the core of the manuscript, other spatial related variables could be included like environmental variables, road structure variables, distance to relevant points (i.e. cities).
If possible, the inclusion of more socio-cultural variables as etnias, level of education could reveal relevant associations.
Given spatial dependency was observed, to a proper analysis and to extract the fitted values of the predictive variables, the model should include in its structure the effect of the spatial dependency. Please revised the use of Conditional autoregressive (CAR) priors, or the Bayesian spatial modelling with INLA, both methods are suggested in this kind of analyses. Also, a spatial model with INLA would allow to perform a predictive model better than Krigging.
Even my native language is not English, I notice that throughout the text are observed sentences with none sense or redundant information, and also grammatical errors. Major revision of text should be performed to get a clear and easy going narrative.
Also, many references do not correspond to the cited data

**Authors:** Thank you very much, dear!!Your concerns are accepted proudly. As much as possible, we have made manuscript clear. We have conducted this study based on available important variable from EMDHS data. As long as the data source is mini version, important variable that might determine households’ access to sanitation service are not collected, and some have missed value. We have written this issue as limitation of the manuscript. Thank you very much again!!
**Specific comments:**
Reviewer #2: Line 16-20: In the abstract, I suggest to avoid mentioning softwares and focus on mentioning the methodology. There are methods not included in the abstract.

**Authors:** Thank you so much! We have removed figures from the abstract section of the manuscript, and important methods are included.

**Authors:** Thank you for your direction,
Reviewer #2: line 24: avoid referring to figures in the abstract.

**Authors:** Thank you so much! We have removed figures from the abstract section of the manuscript.
Reviewer #2: lines 25-27: The inclusion of the sense of the association, so the reader easily understands the findings

**Authors:** Thank you so much! Improvements have been made to it.

Reviewer #2: Line 28: That households have limited sanitation service access it is not a conclusion of the current work, it is widely known and explained in the background section.

**Authors:** Thank you so much! Since the topic is about sanitation service access among households. According to the current finding, 19.7% of households had access to sanitation service. As the figure indicate, it is low or limited. So, we have concluded it as insufficient.
Reviewer #2: Lines 28-30: The conclusion is not in sense on the original research question. Also, focusing on the awareness of residents about this problem, places the responsibility on residents. For what I understood from the manuscript, the main outcome of the study is to identify areas with higher prevalence of lack of access to sanitation services and the associated factors. The conclusion should reflect the main conclusions from the obtained results, and the implications of it.

**Authors:** Thank you so much! We have made an improvement in the conclusion section of the manuscript.
Reviewer #2: Lines 39-40: The sentence presents data as global information, but the references are from local data. Please rephrase the sentences so the reader can understand if is global or local data. If is global data, so the references should be improved.

**Authors:** Thank you so much for your concern! The information has been taken from WHO, and the local information has been rephrased, now.
Reviewer #2: Line 48: please explain if Jimma is a state, municipality, town, so de reader can understand.

**Authors:** Thank you for your concern! Jimma is on town in Ethiopia. So, we have made improvement on it in line with your direction.

Reviewer #2: Line 48: “45% of available latrine facilities are poor in hygiene, and the pipelines are poor in function [12]”. Where?

**Authors:** Thank you for the specific direction! This source where from a study done in Negele town. So, improvements have been made in it in the main revised manuscript.
Reviewer #2: Line 56: which feature of the household head?

**Authors:** Thank you for the specific direction! This was to say the sex of the household head. The correction has been made now.
Reviewer #2: Line 57: Toilet age?

**Authors:** Yes dear! This is about how many years or times the available toilet has been giving service/ How long the toilet has been constructed? We hope that you have got the message.
Reviewer #2: Lines 57-58: rephrase to: “may represent associated factors to sanitation services access”

**Authors:** Thank you for your concern! Corrections have been made in the main revised manuscript.
Reviewer #2: Lines 58-59: please rephrase, to maybe: "could be useful to localize specific areas with greater deprivation of sanitation access where prioritize sanitation interventions.

**Authors:** Thank you for your concern! Corrections have been made in line with your direction.
Reviewer #2: Line 60: Please replace “highlight” to “describe”.

**Authors:** Thank you! We have replaced it.
Reviewer #2: Lines 74-79: The sampling design is not completely understandable. So, I went to read the EMDHS. I notice that many important details are missing here. So, the text should be extended and revised or derived directly from the reference.

**Authors:** Thank you for your concern! Since this study is based on secondary data source which is collected by Ethiopian Public Health Institute in collaboration with the Central Statistical Agency. They used a two-stage stratified sampling procedure to draw the data from respondents at the household level. So, the sampling procedure of this study is in line with EMDHHS’s sampling procedure. For the methods section such as study design, data source, sampling procedure, and population, the reference is cited in the study population section of the manuscript before the variable section.
Reviewer #2: Line 76: how were distributed this 305 EA? How many EA are in the 21 stratas?

**Authors:** Thank you for your concern! 305 EAs were distributed in eleven regions 25 EAs from eight regions (8*25=200 EAs), and the remaining 105 EAs were from the large regions such as Amhara, Oromia, and South Nations Nationality and people’s region (35 EAs from each region). The 305 EAs include 93 Urban and 212 Rural areas. So, in 21 sampling strata 305 EAs exist. For more clarification, I kindly recommend you to check the EMDHS report.

Reviewer #2: Line 77: the sample selection was proportional to the population? Is not clear if the results showed are representative of the population.

**Authors:** Thank you for your concern! Yes Exactly! The sampling procedure clearly explains that the proportional allocation was done among the population. Since the data is collected across all regions in Ethiopia, the data have representative nature for the population.

Reviewer #2: Line 83: what do you mean by zero coordinates?

**Authors:** Thank you very much! Zero coordinate means in short when the latitude and longitude are zero. As you know this study is spatial analysis. What spatial analysis differs from normal statistical analysis? Both use attribute data, but the spatial analysis is done based on latitude and longitude files (Coordinates for the specific value of the attributes based on a respondent’s cluster).
Reviewer #2: Line 84: what do you mean by had no proportional access to sanitation? are missing data or what?

**Authors:** Thank you very much! Actually, it was to say undefined proportion. This means that number of households that had access to sanitation service is divided by the total respondents in a cluster because in a cluster there are households who had access to sanitation service and who had no access to sanitation services. In such cases, the number of households that had access to sanitation services in a cluster might be zero. It leads undefine. To perform spatial analysis, we have assumed this first.
Reviewer #2: Line 87: The output variable is at a household level? as yes or not?

- The outcome variable is sanitation services access. So, the outcome is improved and non-improved or poor. That means the households had Access to improved sanitation services access, if the households had access to at least one of the following: flush or pour-flush to a piped sewer system, septic tank, pit latrines, ventilated-improved pit latrines, or pit latrines with a slab or composting toilets. The household had non-improved (poor) sanitation service access, If the household had any of the following toilet types: flush somewhere else; pit latrines without slabs; open pits, buckets; hanging toilets; and open field defecation, including no facility, bush, or field, it is considered poor sanitation service access.
  Reviewer #2: Lines 93-94: how it was measured wealth status?

**Authors:** Thank you for your question! Wealth status of households was generated from wealth status from EMDHS data set. The variable first had five categories such as poorest, poorer, middle, rich and richest. For statistical analysis purpose, we have labelled in to three category such as poor, middle and rich. Poorest and poorer=poor, middle=middle, and rich and richest=rich. Detail about this is presented in the revised manuscript.
Reviewer #2: Line 94: the category “Shared facilities” by its nature, is not correlated with improved sanitation access? Also, in the EMDHS one of the features that classify the kind of sanitation access, which is the objective to include as an independent variable?

**Authors:** Thank you for your comments. Your concern is crucial. I was also surprised about it. By the way, the variable has its own record which differs from improved and unimproved types of toilet facilities in the EMDHS dataset. In poor communities, with limited space the communities have for toilet construction, and in highly populated areas especially in urban areas, people might share the available improved toilet facility. So, this is for resource-limited settings and poor households if the user uses it properly, and if the toilet is well managed and operated. Sharing toilets is good than nothing has or using open filed/bushes as toilets. Our finding also confirms this fact.
Reviewer #2: Line 96: location of toilet facility, is not naturally related to the conditions of poor sanitation service?

**Authors:** Thank you for your concern! This indicates whether the available toilet is placed in their own yard or not. This indicate how much distance the toilet is far from the households’ yard. The source data is EMDHS, and as you know data at national label was collected considering important public health indicators. So, location of toilet facility is collected as one attribute. We wish to include in our analysis and to look its statistically effect for households’ access to sanitation services.
Reviewer #2: Lines 118-124: the output of the variogram and the kriging should be included in result section or as supplementary material.

**Authors:** Dearest, thank you for your concern. This is how we proceed to get the interpolation of unsampled areas. Already, the interpolation file has been provided as supplementary file 5. We have different techniques for interpolation, of these we had used kriging.
Reviewer #2: Line 122: The input data is at the household or cluster level?

**Authors:** Thank you for your concern! The input data is from respondents in the household. So, respondents are nested within households, and households are nested within clusters. Therefore, respondents from the same cluster are more similar than those respondents who were from another cluster. So, correlation might exist within clusters in terms of the outcome of interest.
Reviewer #2: Line 124: move the reference where corresponds

**Authors:** Thank you for your concern! The figure reference has moved before a line and after the previous line fully stop.

Reviewer #2: Lines 125-137: please specify test settings for statistical inference in Satscan. Also, please specify if in the output the overlapping was allowed and justify the decision.

**Authors:** Thank you for direction. As per our information, likelihood ratio test on the basis of the 999 Monte Carlo replications are the best for statistical analysis for most likely clusters. To avoid overlapping of cluster, SaTScan analysis have been run for times to avoid the geographical overlap by setting 40% 30% 20% 10% of population size. But, still overlapped. We have marked it as a limitation of the study.
Reviewer #2: Line 130: The minimum and maximum spatial cluster size was based on what?

**Authors:** Thank you for your concern! The maximum and minimum cluster size can be defined based on percent of population size.
Reviewer #2: Line 139: Is not entirely clear if the model is at the household or at cluster level. And, which distribution family? binomial? Negative binomial?

**Authors:** Thank you for your concern!! The multilevel analysis models were setted at cluster level to alleviate the data dependency nature within clusters. According to autocorrelation repot, the distribution is non-random.
Reviewer #2: Line 144: replace “variation” with “dependency” or correlation.

**Authors:** Thank you for your concern! The correction has been made.
Reviewer #2: Line 146: In the multilevel model, multicollinearity was tested?

**Authors:** Yes! We have checked the multicollinearity using variance inflation factors.
Reviewer #2: Line 147: which was the random factor? the cluster or the community?

**Authors:** Thank you for your concern! We have made correction in the manuscript.
Reviewer #2: Lines 155-163: The sociodemographic characteristics are from the sample population? It is representative of the studied population? What it means “under the rich wealth index” ? that 56.4% were not rich households? So almost 44% were?

**Authors:** Thank you! Since the data is collected at the national level, it has a representative nature. 56.40% of the household were rich. The remaining 23.20% and 20.40% were poor and middle in the wealth index. This issue is clearly presented in table 1.
Reviewer #2: Lines 164-168: please revise the paragraph, in the first sentence you reflect the proportion with improved sanitation, then in the second without sanitation and in the third, you relate percentages of both services referring to improved and unimproved services. Maybe the structure of the paragraph may be: first focus on the improved types of services, and then to the unimproved services.

**Authors:** Thank you so much! We have rearranged the paragraph.
Reviewer #2: Line 171: limit precision to two decimals

**Authors:** Thank you so much! We have limited to two decimals.
Reviewer #2: Line 173: convert to kilometres

**Authors:** Thank you so much! We have converted it.
Reviewer #2: Line 175: This sentence should be in the figures subtitle or legend.

**Authors:** Thank you! We make it as a subtitle in the figure.
Reviewer #2: Line 175: I interpret that the red color points indicate the area the area where households with poor sanitation services access are aggregated or clustered.

**Authors:** Thank you for your direction! We have reinterpreted it in line with your direction.
Reviewer #2: Lines 179-180: why open defecation is now treated as another category?

**Authors:** Thank you for your concern! This was the wrong correction has been made in the revised manuscript.
Reviewer #2: Line 181: again, these results are representative of the study population? or are the sociodemographic features of the sample population? Age of whom? Sex of whom?
The percentage of household with sharing facilities appears inverted in the text.

**Authors:** Deep thanks for your attention in the manuscript! It is to say age and sex of households’ head. Correction on the inverted figure has been done in the manuscript. The data has been collected at national level. Therefore, the findings might have good representativeness s compared to many studies that have done in single institution or communities in a specific area of the country.

Reviewer #2: Line 183-193: In the way that the analysis was conducted it does not bring relevant information. I think that It should be avoided geographical overlapping in the output, stablish a greater minimum size and a smaller maximum size of cluster. Given that by the way that was conducted most of the territory is included in some cluster.

**Authors:** Thank you for your concern! We tried to minimize 50% of the population size. But, no change at all except the last two (the fifth and sixth) clusters were not detected. We did not know up to what the default size (50%) of the population would be reduced to avoid geographical overlap. We have booked this question for research. Or any direction please share us. Thank you very much for this section.

Reviewer #2: Line 184: This sentence should be in the figure´s subtitle.

**Authors:** Thank you. We have improved the subtitle of the figure.
Reviewer #2: Lines 185-186: This is information is related to what? Are Id´s of clusters? It brings some relevant information?

**Authors:** Yes dear, they are the ID’s of the clusters! They have great value to describe the the satscan map.
Reviewer #2: Lines 187-190: is more useful to show this information only in the map as a figure.

**Authors:** Thank you for your insight! The Sat Scan analysis’ result is displayed in map form. For the displayed map, details about the map is realised in text form. To make the map clear, those detail intext form is better if they compiled in table form. Now, we do not have any option to present the detail of the map using table and text.
Reviewer #2: Figure 1: this figure is likely a Moran I method explanation than a result, if relevant, should be in supplementary material. Improve subtitle to be self-explained

**Authors:** Thank you for your comment! Yes dear, it is based on Moran I or spatial autocorrelation analysis. We tried to add subtitle on it as another figures. However, this Figure is an automatic, we could not add anything on it. But, as much as possible clear and explanatory legend has been provided at the end of the reference list. Thank you again.
Reviewer #2: Figure 2: Improve the legend. Why open defections is a different class? Improve region labels to be clearly read. Improve subtitle to be self-explained

**Authors:** Thank you for your concern! Corrections have been made in Figure 2.
Reviewer #2: Figure 3: Improve legend. what it means the value? Improve subtitle to be self-explained.

**Authors:** Thank you for your concern! Corrections have been made in Figure 3.
Reviewer #2: Figure 4: Improve legend.
About the figures in general, please adapt the scale to coincide between the three figures. Also, given the three figures are based on the same map of Ethiopia, they could be converted into one figure where appears the three maps.
**Authors:** Thank you for your concern! Corrections have been made in Figure 4, and all others. I tried to merge the map of all figures in one but, I faced a space problem.
Table 2: Please modify the orientation of the table.

**Authors:** Thank you. We have modified it.
218: “place of residency refers to what?

**Authors:** Thank you! It refers to rural households. The correction has been made about it.
219: rephrase: "to appropriate sanitation service access"

**Authors:** Thank you! We have rephrased it.
224: “staying on-premises” means households that obtain their water on its own premise?

**Authors:** Yes! Improvements have been made to it in line with your direction.
Table 3: improve the table title and explain the response variable

**Authors:** Thank you! Improvements have been made to it in line with your concern.
236: Some data appear different in the EDHS, for example, the percentage of households with access to improved sanitation services in the rural area.

**Authors:** Thank you dear for your concern! The existing difference is not about the sanitation service access. In our analysis, 61.80% does not indicate the rural households’ sanitation service access rather it shows how many households are from rural areas based on the sample. According to the EMDHS report, 20% of households used improved sanitation services of which 10% are in rural areas. So, the difference is not based on sanitation service access.

253-254: It is redundant to say that households in the significant clusters were more likely to be vulnerable to poor sanitation service access than households outside the window.

**Authors:** Thank you! As much as possible, redundant have been reduced.
254-257: Please revise the text. The interpolation only showed the inputted information, didn´t confirmed.

**Authors:** Thank you! We have revised it. Please have a look it in the manuscript.
261: Sharing toilet facilities is a classifier characteristic by the EMDHS to determine if the household has access to improved sanitation services or not, if you treated differently, you should justify and discuss it.

**Authors:** Thank you! Sharing toilet facilities are treated differently. It indicates whether a household shared toilet facility with another households. EMDHS report also treat it as one of the most important public health markers.

275: Please review the sentence, a connector is missing after “might be”.

**Authors:** Thank you for your direction. Corrections have been made there.
282-284: The sentences is repeating information from the previous sentence.

**Authors:** Thank you for concern. Corrections have been made there.
286: what it means with “residency”.

**Authors:** Thank you. This is to say whether the household is from rural or urban.
301-302. Review the sentences, and avoid “poor enough”. Maybe: “rural people may not Count with adequate financial resources and so are unable to access improved sanitation services”

**Authors:** Thank you for your direction. Corrections have been made there.
305-311: Review the paragraph. Avoid repeating results in the conclusion section. Focus on bringing the outputs of the study, relevant information for the stakeholders, and relevant information for global public health academic community.

**Authors:** Thank you for your direction. Corrections have been made in the revised manuscript.
318-319. Rephrase the sentence. “specific vulnerable households spatially” does not have sense.

**Authors:** Thank you. We have made an improvement on it.
